# RESPONDER – diagnosis of pathological complete response by vacuum-assisted biopsy after neoadjuvant chemotherapy in breast Cancer - a multicenter, confirmative, one-armed, intra-individually-controlled, open, diagnostic trial

**DOI:** 10.1186/s12885-018-4760-4

**Published:** 2018-08-25

**Authors:** Joerg Heil, Peter Sinn, Hannah Richter, André Pfob, Benedikt Schaefgen, André Hennigs, Fabian Riedel, Bettina Thomas, Marc Thill, Markus Hahn, Jens-Uwe Blohmer, Sherko Kuemmel, Maria Margarete Karsten, Mattea Reinisch, John Hackmann, Toralf Reimer, Geraldine Rauch, Michael Golatta

**Affiliations:** 1 Department of Gynecology, Breast Center, Heidelberg University, Heidelberg, Germany; 2 Department of Pathology, Heidelberg University, Heidelberg, Germany; 3 Koordinierungszentrum für Klinische Studien (KKS), Heidelberg University, Heidelberg, Germany; 4 Department of Gynecology, Agaplesion Markus Hospital, Frankfurt am Main, Germany; 5 Department of Gynecology, Tuebingen University, Tuebingen, Germany; 6 Department of Gynecology, Charité Universitaetsmedizin Berlin, Berlin, Germany; 7 Department of Gynecology, Hospital Kliniken Essen-Mitte, Essen, Germany; 8 Department of Gynecology, Marien Hospital Witten, Witten, Germany; 9 Department of Gynecology, Rostock University, Rostock, Germany; 10 Charité Universitaetsmedizin Berlin, Institute of Biometry and Clinical Epidemiology, corporate member of Freie Universität Berlin, Humboldt-Universität zu Berlin, and Berlin Institute of Health Berlin, Berlin, Germany; 11 Berlin Institute of Health (BIH), Berlin, Germany; 12 Institute of Medical biometry and Informatics, Heidelberg University, Heidelberg, Germany

**Keywords:** Breast cancer, Neoadjuvant chemotherapy, Treatment response evaluation, Vacuum-assisted biopsy

## Abstract

**Background:**

Neoadjuvant chemotherapy (NACT) is a standard approach of the multidisciplinary treatment of breast cancer. Depending on the biological subtype a pathological complete response in the breast (bpCR) can be achieved in up to 60% of the patients. However, only limited accuracy can be reached when using imaging for prediction of bpCR prior to surgery. Due to this diagnostic uncertainty, surgery after NACT is considered to be obligatory for all patients in order to either completely remove residual disease or to diagnose a bpCR histologically. The purpose of this trial is to evaluate the accuracy of a vacuum-assisted biopsy (VAB) to diagnose a bpCR after NACT prior to surgery.

**Methods:**

This study is a multicenter, confirmative, one-armed, intra-individually-controlled, open, diagnostic trial. The study will take place at 21 trial sites in Germany. Six hundred female patients with breast cancer after completed NACT showing at least a partial response to NACT treatment will be enrolled. A vacuum-assisted biopsy (VAB) guided either by ultrasound or mammography will be performed followed by histopathological evaluation of the VAB specimen before standard, guideline-adherent breast surgery. The study is designed to prove that the false negative rate of the VAB is below 10%.

**Discussion:**

As a bpCR is becoming a more frequent result after NACT, the question arises whether breast surgery is therapeutically necessary in such cases. To study this subject further, it will be crucial to develop a reliable test to diagnose a bpCR without surgery.

During the study we anticipate possible problems in patient recruitment as the VAB intervention does not provide participating patients with any personal benefit. Hence, a proficient informed consent discussion with the patient and a detailed explanation of the study aim will be crucial for patient recruitment. Another critical issue is the histopathological VAB evaluation of a non-tumorous specimen as this may have been taken either from the former tumor region (bpCR) or outside of the (former) tumor region (non-representative VAB, sampling error).

**Trial registration:**

The trial has been registered at clinicaltrials.gov with the identifier NCT02948764 on October 28, 2016 and at the German Clinical Trials Register (DRKS00011761) on February 20, 2017. The date of enrolment of the first participant to the trial was on March 8, 2017.

## Background

Neoadjuvant chemotherapy (NACT) is a standard part of the multidisciplinary treatment of breast cancer [1]. Nowadays up to 30% of all breast cancer patients receive NACT [2]. NACT has been shown to be equivalent to adjuvant chemotherapy in terms of disease free, distant disease-free, and overall survival in several clinical trials and enables more breast cancer patients to receive breast-conserving therapy [3–6]. Depending on the biological subtype of the tumor, up to 60% of the patients achieve a pathological complete response in the breast (bpCR) [7, 8]. The most conservative definition of a bpCR was found to be a complete disappearance of invasive and in situ residual tumor disease in the breast (ypT0) [9–11].

Achieving a bpCR is a predictor for an improved disease free and overall survival, and it is used as a surrogate clinical endpoint for long term outcome [1, 4, 6, 10–16]. Only mediocre diagnostic accuracy can be reached when predicting a bpCR before surgery by a combination of multiple aspects such as tumor biology, the applied NACT regimen, and breast imaging results [8, 17–20].

Due to this diagnostic uncertainty, surgery after NACT is considered to be obligatory for all patients in order to either completely remove residual disease in non-bpCR cases or to diagnose a bpCR [21]. So far, surgery is the only valid diagnostic instrument to diagnose a bpCR. However, there is evidence that in cases of a shrinking tumor a less radical breast surgery is oncologically safe. [22]. The case of a clinical or imaging complete response (cCR), however, requires the diagnostic resection of (parts of) the initial tumor bed in order to confirm (or not) a possible bpCR histologically [6, 23].

## Methods/design

### Aims

The main purpose of this trial is to evaluate the accuracy of a vacuum-assisted biopsy (VAB) for a reliable diagnosis of a bpCR after NACT. The study is designed to prove the false negative rate of the VAB is below 10% (= sensitivity is 90% or above).

### Design

This study is designed as a multicenter, confirmative, one-armed, intra-individually-controlled, open, diagnostic trial. Patients will be recruited in 21 centers in Germany.

### Participants

Participants are female patients aged 18 years and older with primary breast cancer after NACT treatment which has been performed for at least 12 weeks and resulted in cPR or cCR (see below). Patients can be enrolled if the following inclusion criteria are met: any cT and cN stage, except cT4 stages; patient is scheduled to undergo any routine breast cancer surgical intervention planned according to guidelines (breast conservation or mastectomy); the residual intramammary target lesion or clip marker is visible in mammography and / or ultrasound; diagnosis of imaging complete or partial response according to RECIST 1.1 by mammography or ultrasound, according to local routine; in case of multicentric disease: confirmation of the same tumorbiological subtype of tumor defined by immunohistology in at least 2 lesions. Only one breast per patient will be included, in bilateral cancer one breast can be included. Patients have to be able to understand the character and individual consequences of the clinical trial and must give written informed consent before enrollment in the trial. Patients will be excluded from the trial in case of palliative or recurrent breast cancer. Further exclusion criteria are dislocation of clip marker (> 5 mm distance to the initial target lesion border at the time of clip placement), contraindication for VAB or associated procedures (e.g. local anesthesia) as well as pregnancy and lactation.

### Intervention

In this study design the control (breast surgery = reference test) and the comparator (VAB = index test) will both be performed on every patient. After an initial screening visit (visit 1), during which inclusion criteria will be checked and informed consent will be obtained, the VAB will be performed (visit 2). This intervention visit may vary by patient, tumor, and trial site characteristics and may either be an ultrasound guided or a stereotactically guided VAB. In analogy to the German S3 guideline on primary breast cancer management, we recommend to take at least 12 biopsies with 10G needles or less in case of larger needle sizes [24], bearing in mind that the probability of a sampling error might be reduced by taking more samples. As quantification of the specimen is not easily possible during the VAB (weight, as well as the number of biopsies taken does not necessarily quantify the amount of adequate tissue), we propose to take as many samples as reasonably justifiable (according to the local investigator). The VAB will be performed according to standards in primary breast diagnostics and according to the above mentioned guidelines. The intervention may be performed before surgery at a separate visit, the day before surgery (e. g. during the wire location), or in the operation room immediately prior to surgery depending on the site specific organizational setting. As the surgery will be performed according to clinical routine, there is no specific time frame for each trial visit. The physician performing the biopsy will be asked to quantify subjectively the level of representativeness of the biopsy (secondary outcome measure). The imaging performed during VAB may not be used for assessment of inclusion/exclusion criteria. Specimen radiography may be applied as an optional procedure after VAB to assess the representativeness of the specimen radiologically (secondary outcome measure). Standard surgery is regarded as the third trial visit (visit 3) as standard surgical excision (either breast conserving surgery or mastectomy) is the reference test. Adverse events will be documented until the end of visit 3.

### Adverse events

Possible adverse events of the VAB procedure may occur while the biopsy is taken. Due to the simple study design following clinical routine, very few adverse events are expected. Within the pilot study [25] there were no safety issues. As the biopsy is an additional minimal invasive intervention, it is accompanied by a number of possible risks. Those include bleeding with (1) hematoma and (2) possible urgent surgical intervention, infection, and injuries of surrounding tissue. Theoretically, VAB could challenge the surgeon, e. g. due to a hematoma and may lead to limitations in reliably assessing (3) the final tumor size or (4) the resection margins by pathology. The first two are informative but not critical; the latter situations are also possible even without preoperative VAB but should not exceed 10% of the patients (ypTx </= 10% and final R1 / Rx status </= 10%). We propose to interrupt the study as soon as those cases exceed 10% of the whole cohort.

### Intervention assignment and blinding

There will be no randomization within this one-armed study design.

To be able to transfer the results to future management concepts (e. g. omitting surgery in cases of VAB – proven bpCR) the histopathological, study specific evaluation of the VAB specimen will be performed independently of the routine diagnostic evaluation. Nevertheless, the local pathologist will have access to information on pre-NACT histopathological result, cT stage, ycT stage, estrogen receptor status, progesterone receptor status, Her2neu status, grading, and ki-67 status (if available). However, she / he will not receive any information regarding post-NACT surgical specimen results. All pre-surgical variables should be available and included in the pathological evaluation of the VAB specimen after NACT. The interdisciplinary team, including the breast surgeon as well as the patient, will also be blinded to pathological results of the VAB specimen until the final pathological report of the surgical specimen. This will ensure an unbiased surgical intervention.

Pathological work-up of VAB specimens will be performed in analogy to the primary diagnostic setting of suspicious breast lesions [26]. In order to achieve consistency among pathologists regarding the criteria for evaluation of the VAB specimen, a standard operating procedure will be provided as well as a the possibility of using a reference pathologist’s second opinion.

The pathological results of the VAB specimen will be categorized as follows:

**Table:** 

Category A	Residual tumor cells in VAB specimen (= non-bpCR)
Category B	No residual tumor cells in the VAB specimen and VAB representative of former tumor region (= bpCR in VAB)
Category C	No residual tumor cells in the VAB specimen but VAB unclear representative or not representative of former tumor region (= possible sampling error)

Category C VABs are categorized as uninformative for the primary endpoint of the clinical trial.

The investigators assume that there is no difficulty to diagnose a “Category A” VAB result. The challenging topic is defining a VAB specimen to be pathologically representative of the former tumor region (tumor bed) or not, i. e. to differentiate between “Category B” and “Category C”. Only representative VAB samples are informative.

To evaluate intra- and interrater reliability of pathological evaluation of VAB specimen all local pathologists will be asked to send pre-NACT biopsy specimen and post-NACT VAB specimen (H&E sections or virtual microscopy files) of all non-tumorous VABs to the Department of Pathology Heidelberg (“pathological sub-study”). Slides will be digitized, pseudonymized, and returned to different local pathologists. Intra- and interrater reliability evaluation will be performed by at least five pathologists participating in the trial.

### Outcomes and measurements

The primary outcome is the false negative VAB result, i.e. the non-detected residual tumor by VAB compared to breast surgery. We will report this outcome as the false negative rate (FNR = rate of patients with non-detected residual tumor by VAB compared to breast surgery) which is a commonly used and validated measure in diagnostic studies. The FNR will serve as outcome measure in the cohort of the confirmatory primary outcome analysis. It will be calculated as the quotient of the number of cases with bpCR in VAB (“Category B”) and residual tumor in surgical specimen (false negative VAB results), divided by the total number of cases with residual tumor in either specimen (VAB and / or surgical specimen). Residual tumor is defined as a positive result, in surgical specimen as well as in VAB.

As our secondary outcomes we will use true negative and true positive results compared to breast surgery. The standard definitions are applied.

### Statistical procedures

#### Sample size calculation

As the primary endpoint we use the rate of patients with non-detected residual tumor (=false negative cases), the sample size calculation refers to the required number of patients for which surgery revealed a residual tumor. This number depends on the prevalence of residual tumor in the whole cohort. Motivated by previous works and the definition of the inclusion and exclusion criteria, we assume that this prevalence is at least 0.5. For the test hypotheses the sample size required to achieve a power of 0.8 is given by 238 cases (calculated with the software ADDPLAN Version 6.1.1). Consequently, a total of 476 patients have to be recruited to reach the required number of cases with residual tumor. As the prevalence of 0.5 is only an estimator and, moreover, some patients may be excluded from the analysis because of an unclearly representative VAB, we add an exclusion rate of 0.25 according to the findings in the pilot study [25]. This results in a total number of 595 patients, which will be rounded up to 600 patients, to be assigned to the trial.

### Analysis variables

#### Derivation of the primary endpoint

The false negative rate (FNR) will be calculated as the quotient of the number of cases with “pCR in VAB” and residual tumor in surgical specimen (false negative VAB results), divided by the total number of cases with residual tumor in surgical specimen and / or VAB in the cohort of the confirmatory primary outcome analysis (= primary analysis set).

#### Derivation of secondary endpoints

Specificity, negative, and positive predictive values will be calculated according to the standard definitions.

#### Primary analysis set

This data set defines the cohort of patients based on which the primary confirmatory efficacy analysis is performed. The primary analysis set consists of all recruited patients excluding those with a pathologically defined uninformative VAB sample tissue (“Category C”: unclear representative or not representative VAB).

#### Secondary analysis set

The Secondary Analysis set consists of all recruited patients including those with an unclear representative or not representative VAB sample tissue in the histopathological assessment. This data set defines the cohort of patients and subsamples based on which the secondary descriptive analyses are performed (see below “Secondary Analysis”).

### Analysis strategy

#### Primary efficacy analysis

The null hypothesis to be assessed within the confirmatory analysis states that the rate of patients with non-detected residual tumor p is larger or equal to 0.1. Whereas the alternative hypothesis, for which this trial is powered, states that p is at most 0.05. The test hypotheses are thus given by.

H0: *p* ≥ 0.1 versus H1: *p* < 0.05,

which are tested with the one-sample Binomial-test at a one-sided significance level of α = 0.025.

The rate of patients with non-detected residual tumor p is estimated by the number of cases with diagnosed bpCR in VAB divided by the number of all patients for which surgery or VAB revealed a residual tumor.

#### Secondary analyses

All secondary endpoints are evaluated descriptively for the Primary Analysis Set (all recruited patients excluding “Category C”) as well as the Secondary Analysis Set (all recruited patients including “Category C”) in order to allow comparability and evaluation of the histopathological analysis. The secondary endpoints will be evaluated for the whole cohort and for six subgroups defined by.

1. the tumor biology (TNBC, HER2+, and HR+/HER2-)
2. the clinical / imaging response assessment (cCR, near cCR, cPR)
3. the assessment of representativeness by specimen radiography.
4. the subjective rating of the physician performing the biopsy.
5. the different hospitals taking part in the study.
6. the guidance method of the minimal invasive biopsy (ultrasound / stereotactic)

The investigators will provide point estimators and corresponding 95% confidence intervals for sensitivity, specificity, and diagnostic odds ratios for bpCR. Predictive values are obtained by using Bayes’ theorem based on the different prevalence estimates for bpCR in the full cohort, the subgroups mentioned above, and the trial centers.

The pathological evaluation of the VAB specimen (“pathological sub-study”, see above) will be assessed using Cohen’s Kappa where satisfactory agreement will be defined as an observed value of > / = 0.7.

Absolute and relative frequencies of adverse events are provided together with 95% confidence intervals.

We plan to perform an interim data look after having recruited and fully documented 300 participants. Within this interim look, no early testing for efficacy will be performed. However, it will be evaluated if a stop for futility seems indicated. A stop for futility could be indicated if the number of false negative cases is too large to reach the study aim (non-stochastic curtailment) or for other safety reasons.

## Discussion

To date, there are a number of closed, ongoing, and planned trials on this subject, described elsewhere in detail [27]:

As we do not know the maximum sampling error (= FNR) without impairing loco-regional control rates in case of omitting surgery, we deduced a maximum FNR of 10% from the results of the sentinel node trials. In these studies a false negative rate of 10% did not translate into a worse loco-regional or overall survival [28]. More recently, the ACOSOG Z0011 trial showed, that leaving lymph node metastases behind in about 20% of the cases, did not translate into worse loco-regional, disease-free and overall survival [29]. As the cohort of patients treated with NACT is a high risk population we decided for a reasonably low FNR.

During the study we anticipate a set of possible challenges in patient recruitment. First of all, the VAB intervention does not provide participating patients with a personal benefit. By contrast, it involves a second invasive intervention in addition to surgery. We thus assume patients to be reluctant to participate in the study. Hence, a proficient informed consent discussion with the patient and a detailed explanation of the study aim will be crucial for patient recruitment.

Another critical issue in VAB evaluation will be the histopathological evaluation of the representativeness of the VAB specimen. Differentiating accurately between a Category B (= pCR in VAB) and Category C (= possible sampling error) result will be decisive for the achievement of qualified study results. In the pilot study [25] anticipating this RESPONDER trial, the following stroma and cell reactions were used as criteria based on preceding reports [30–32]. Typical stroma reactions due to NACT included oedematous swelling or fibrosis. Within the fibrotic area a low cell-density was found. Stroma cell reactions were constituted of atypical adenoid cell complexes with large nuclei, regressively transformed residuals of atypical ductal epithelial proliferation, atypical ductal epithelial hyperplasia, or metaplasia of cylindrical cells. Furthermore, cell reactions included macrophage reactions such as the presence of foam cell-like macrophages, giant cells, hemosiderin-charged macrophages, or foam cells indicating resorptive processes. Round cell infiltrates and lympho-histiocytic inflammatory infiltrates were partly present. Based on these findings and experiences histopathological evaluation should allow a standardised diagnostic categorisation.

In case of study results which would permit rejection of the null hypothesis by yielding a false negative rate of less than 10%, VAB would be rated as an accurate diagnostic measure. Possible future trial scenarios would raise difficult questions: In this still hypothetical setting, studies on the therapeutical impact of surgery in bpCR cases would be the consistent consequence. However, the possible setting in which such a study could be realized remains unclear. Ethical and technical issues will have to be addressed for future trial designs. One future option might be a two-armed randomized controlled trial with radiotherapy only as local therapeutic management as one arm and surgery (with or without radiotherapy according to guidelines) as the other arm. Potential endpoints of such a trial might be disease free or in-breast recurrence free survival. Recruitment may be a challenge in a trial offering a non-surgical treatment arm. However, sooner or later the planning of possible future clinical trials on the omission of surgery in bpCR cases will have to be discussed.

## Abbreviations

bpCR: Pathological compete response in the breast
cCR: Clinical complete response
cPR: Clinical partial response
DFG: Deutsche Forschungsgemeinschaft (German Research Foundation)
EC: Ethics committee
FNR: False negative rate
ITT: Intention to treat
NACT: Neoadjuvant chemotherapy
NPV: Negative predictive value
pCR: Pathological compete response
PP: per-Protocol
PPV: Positive predictive value
VAB: Vacuum-assisted biopsy

## References

[1] Kaufmann M, Hortobagyi GN, Goldhirsch A, Scholl S, Makris A, Valagussa P, Blohmer JU, Eiermann W, Jackesz R, Jonat W (2006). Recommendations from an international expert panel on the use of neoadjuvant (primary) systemic treatment of operable breast cancer: an update. J Clin Oncol.

[2] Hennigs A, Riedel F, Marme F, Sinn P, Lindel K, Gondos A, Smetanay K, Golatta M, Sohn C, Schuetz F (2016). Changes in chemotherapy usage and outcome of early breast cancer patients in the last decade. Breast Cancer Res Treat.

[3] Mauri D, Pavlidis N, Ioannidis JP (2005). Neoadjuvant versus adjuvant systemic treatment in breast cancer: a meta-analysis. J Natl Cancer Inst.

[4] Fisher B, Bryant J, Wolmark N, Mamounas E, Brown A, Fisher ER, Wickerham DL, Begovic M, DeCillis A, Robidoux A (1998). Effect of preoperative chemotherapy on the outcome of women with operable breast cancer. J Clin Oncol.

[5] Untch M, von Minckwitz G: Neoadjuvant chemotherapy: early response as a guide for further treatment: clinical, radiological, and biological. J Natl Cancer Inst Monogr 2011, 2011(43):138–141. 10.1093/jncimonographs/lgr028 22043061

[6] Kaufmann M, von Minckwitz G, Mamounas EP, Cameron D, Carey LA, Cristofanilli M, Denkert C, Eiermann W, Gnant M, Harris JR (2012). Recommendations from an international consensus conference on the current status and future of neoadjuvant systemic therapy in primary breast cancer. Ann Surg Oncol.

[7] Rody A, Karn T, Solbach C, Gaetje R, Munnes M, Kissler S, Ruckhaberle E, Minckwitz GV, Loibl S, Holtrich U (2007). The erbB2+ cluster of the intrinsic gene set predicts tumor response of breast cancer patients receiving neoadjuvant chemotherapy with docetaxel, doxorubicin and cyclophosphamide within the GEPARTRIO trial. Breast.

[8] Goldstein NS, Decker D, Severson D, Schell S, Vicini F, Margolis J, Dekhne NS (2007). Molecular classification system identifies invasive breast carcinoma patients who are most likely and those who are least likely to achieve a complete pathologic response after neoadjuvant chemotherapy. Cancer.

[9] Chevallier B, Roche H, Olivier JP, Chollet P, Hurteloup P (1993). Inflammatory breast cancer. Pilot study of intensive induction chemotherapy (FEC-HD) results in a high histologic response rate. Am J Clin Oncol.

[10] van der Hage JA, van de Velde CJ, Julien JP, Tubiana-Hulin M, Vandervelden C, Duchateau L (2001). Preoperative chemotherapy in primary operable breast cancer: results from the European Organization for Research and Treatment of Cancer trial 10902. J Clin Oncol.

[11] von Minckwitz G, Untch M, Blohmer JU, Costa SD, Eidtmann H, Fasching PA, Gerber B, Eiermann W, Hilfrich J, Huober J (2012). Definition and impact of pathologic complete response on prognosis after neoadjuvant chemotherapy in various intrinsic breast cancer subtypes. J Clin Oncol.

[12] Wolmark N, Wang J, Mamounas E, Bryant J, Fisher B. Preoperative chemotherapy in patients with operable breast cancer: nine-year results from National Surgical Adjuvant Breast and bowel project B-18. J Natl Cancer Inst Monogr. 2001;(30):96–102. 10.1093/oxfordjournals.jncimonographs.a003469 11773300

[13] Kurosumi M (2004). Significance of histopathological evaluation in primary therapy for breast cancer--recent trends in primary modality with pathological complete response (pCR) as endpoint. Breast Cancer.

[14] Kuerer HM, Newman LA, Smith TL, Ames FC, Hunt KK, Dhingra K, Theriault RL, Singh G, Binkley SM, Sneige N (1999). Clinical course of breast cancer patients with complete pathologic primary tumor and axillary lymph node response to doxorubicin-based neoadjuvant chemotherapy. J Clin Oncol.

[15] Bear HD, Anderson S, Smith RE, Geyer CE, Mamounas EP, Fisher B, Brown AM, Robidoux A, Margolese R, Kahlenberg MS (2006). Sequential preoperative or postoperative docetaxel added to preoperative doxorubicin plus cyclophosphamide for operable breast cancer:National Surgical Adjuvant Breast and bowel project protocol B-27. J Clin Oncol.

[16] Schwartz GF, Hortobagyi GN, Masood S, Palazzo J, Holland R, Page D, Consensus Conference C (2004). Proceedings of the consensus conference on neoadjuvant chemotherapy in carcinoma of Breast, April 26–28, 2003, Philadelphia, PA. Hum Pathol.

[17] von Minckwitz G, Schneeweiss A, Loibl S, Salat C, Denkert C, Rezai M, Blohmer JU, Jackisch C, Paepke S, Gerber B (2014). Neoadjuvant carboplatin in patients with triple-negative and HER2-positive early breast cancer (GeparSixto; GBG 66): a randomised phase 2 trial. Lancet Oncol.

[18] Shin HJ, Kim HH, Ahn JH, Kim SB, Jung KH, Gong G, Son BH, Ahn SH (2011). Comparison of mammography, sonography, MRI and clinical examination in patients with locally advanced or inflammatory breast cancer who underwent neoadjuvant chemotherapy. Br J Radiol.

[19] Schneeweiss A, Chia S, Hickish T, Harvey V, Eniu A, Hegg R, Tausch C, Seo JH, Tsai YF, Ratnayake J (2013). Pertuzumab plus trastuzumab in combination with standard neoadjuvant anthracycline-containing and anthracycline-free chemotherapy regimens in patients with HER2-positive early breast cancer: a randomized phase II cardiac safety study (TRYPHAENA). Ann Oncol.

[20] Schaefgen B, Mati M, Sinn HP, Golatta M, Stieber A, Rauch G, Hennigs A, Richter H, Domschke C, Schuetz F (2016). Can routine imaging after neoadjuvant chemotherapy in breast Cancer predict pathologic complete response?. Ann Surg Oncol.

[21] Early and Locally Advanced Breast Cancer: Diagnosis and treatment. In: NICE Clinical Guidelines, No 80. Edn. Cardiff (UK): National Collaborating Centre for Cancer (UK); 2009. 20704053

[22] Kaufmann M, Morrow M, von Minckwitz G, Harris JR, Biedenkopf Expert Panel M (2010). Locoregional treatment of primary breast cancer: consensus recommendations from an international expert panel. Cancer.

[23] Wockel A, Kreienberg R (2008). First revision of the German S3 guideline ‘Diagnosis, therapy, and follow-up of breast Cancer. Breast Care (Basel).

[24] Preibsch H, Baur A, Wietek BM, Krämer B, Staebler A, Claussen CD, Siegmann-Luz KC (2013). Vakuumbiopsie der Brust mit 7Gauge-, 8Gauge-, 9Gauge-, 10Gauge- und 11Gauge-Nadeln – wie viele Biopsate sind notwendig?. Senologie - Zeitschrift für Mammadiagnostik und -therapie.

[25] Heil J, Schaefgen B, Sinn P, Richter H, Harcos A, Gomez C, Stieber A, Hennigs A, Rauch G, Schuetz F (2016). Can a pathological complete response of breast cancer after neoadjuvant chemotherapy be diagnosed by minimal invasive biopsy?. Eur J Cancer.

[26] Hahn M, Krainick-Strobel U, Toellner T, Gissler J, Kluge S, Krapfl E, Peisker U, Duda V, Degenhardt F, Sinn HP (2012). Interdisciplinary consensus recommendations for the use of vacuum-assisted breast biopsy under sonographic guidance: first update 2012. Ultraschall Med.

[27] Kuerer HM, Vrancken Peeters M, Rea DW, Basik M, De Los Santos J, Heil J (2017). Nonoperative Management for Invasive Breast Cancer after Neoadjuvant Systemic Therapy: conceptual basis and fundamental international feasibility clinical trials. Ann Surg Oncol.

[28] Krag DN, Anderson SJ, Julian TB, Brown AM, Harlow SP, Costantino JP, Ashikaga T, Weaver DL, Mamounas EP, Jalovec LM (2010). Sentinel-lymph-node resection compared with conventional axillary-lymph-node dissection in clinically node-negative patients with breast cancer: overall survival findings from the NSABP B-32 randomised phase 3 trial. Lancet Oncol.

[29] Giuliano AE, Hunt KK, Ballman KV, Beitsch PD, Whitworth PW, Blumencranz PW, Leitch AM, Saha S, McCall LM, Morrow M (2011). Axillary dissection vs no axillary dissection in women with invasive breast cancer and sentinel node metastasis: a randomized clinical trial. JAMA.

[30] Sinn HP, Schmid H, Junkermann H, Huober J, Leppien G, Kaufmann M, Bastert G, Otto HF (1994). Histologic regression of breast cancer after primary (neoadjuvant) chemotherapy. Geburtshilfe Frauenheilkd.

[31] Provenzano E, Bossuyt V, Viale G, Cameron D, Badve S, Denkert C, MacGrogan G, Penault-Llorca F, Boughey J, Curigliano G (2015). Standardization of pathologic evaluation and reporting of postneoadjuvant specimens in clinical trials of breast cancer: recommendations from an international working group. Mod Pathol.

[32] Fan F (2009). Evaluation and reporting of breast cancer after neoadjuvant chemotherapy. Open Pathol J.

